# Obstetrics and Gynecology

**Published:** 1903-11

**Authors:** Maud J. Frye

**Affiliations:** Buffalo, N. Y


### PROGRESS IN MEDICAL SCIENCE.

### Obstetrics and Gynecology.

Conducted by MAUD J. FRYE, M. D., Buffalo, N. Y.

### LONG-CONTINUED FUNCTION OF THE MILK GLANDS.

Nussbaum (*Munchener Medicinische Wochenschrift*) reports in his practice two cases of persistent secretion of milk for one or more years after the discontinuance of nursing, resulting in making the woman weak and nervous. Both patients were suffering from malposition of the uterus, accompanied with endometritis and fluor albus. The treatment in both was similar, consisting of a pressure bandage over the gland, the use of a mild aperient, and repeated doses of antipyrin to the limit. This soon caused a cessation of the milky secretion with an improvement of the accompanying symptoms. The writer quotes another case from Zeitlin in which the function of the milk glands persisted for five years; the interesting feature in this case was the atrophy of the uterus after a dry metritis associated with amenorrhea.—Abstract in *American Medicine.*

---

THE IODINE TREATMENT OF PUERPERAL SEPSIS.

PRYOR (*New York Med. Jour.,* August 22, 1903.) relates the results in thirty-seven cases operated on by him for puerperal sepsis. Twenty-seven had not been previously operated upon. Of these one died. Ten had been cureted and of these three died. In 36 of the 37 cases streptococci, generally mixed with other germs were found in the uterine cavity, and in all the cases streptococci were found in the serum or lymph, or free pus in the cul de sac. Pryor's method consists in cureting the infected uterus and opening broadly the posterior cul de sac, packing both with iodoform gauze. The almost uniformly favorable result secured he believes to be due to the liberation of free iodine from the iodoform gauze. The sterilisation of the pelvis was demonstrated to take place by the second or third dressing and the urine showed the presence of iodine in the circulation. In all cases either enteroclysis or intravenous infusion of normal salt solution was practised.

---

THYROIDS IN MAMMARY INACTIVITY.

BARNES (*Medical News,* October 10, 1903,) in an article on advances in therapeutics in pediatric practice, relates several cases in which the administration of thyroid extract before confinement and during lactation resulted in an abundant milk secretion in women who following previous confinements had been unable to nurse their offspring.

---

DANGERS OF UTERINE CURETMENT.

VAN DE WARKER (*New York Medical Jour.,* October 10, 1903,) emphasises by the recital of a number of disastrous cases which he has seen in consultation practice, the dangers of uterine curetment. He dwells upon the need for thorough cleansing of the vagina and vulva. He regards Sims's position as the best for the patient, as in it the vagina is ballooned out and can be perfectly cleansed. He cautions against the use of the branching dilator and advises instead the graduated sounds. The sharp curet he regards as dangerous as a high explosive. In the majority of cases he believes the dull curet answers every purpose. The irrigating curet he condemns. Instead of the irrigation he advises wiping the uterus out with sterile cotton. Firm packing of the uterine cavity should never be done. Nothing more than a slender strip of gauze should ever be used.

---

### BACTERIOLOGY OF THE PUERPERAL UTERUS.

MARX (*American Jour. of Obstetrics,* September, 1903,) discusses the following problems: (1) Is the puerperal uterus a sterile organ? (2) In what way does a uterus free from bacteria influence or assist us in diagnosticating a non-septic condition? (3) As a result of these investigations how is our treatment of the parturient state to be influenced?

Fifteen puerperal women were examined by means of culture tubes, 48 bacteriologic tests being made altogether, the observations being begun on the day of labor and extending through the first 5 or 6 days of the puerperium. He concludes that the puerperal uterus is a sterile organ. The presence of bacteria in the puerperal uterus in the absence of general evidence of a constitutional disturbance, such as fever and pulse rise, and the like, means the introduction of such bacteria by accidental contamination.

The presence of bacteria in the puerperal uterus accompanied by fever, rapid pulse and other disturbances means in all probability a sepsis arising from the uterus. The absence of bacteria in the presence of general symptoms indicates the necessity of looking for the source of the disturbance in some organs other than the uterus—sepsis from vagina or vulva or some other general disturbance independent of the puerperal condition. Practically he feels that a systematic examination of the contents of uterus from the standpoint of bacteriology is a very useful procedure, whose field of application is very small—limited to those few cases in which one is unable to make a diagnosis by other means, and further limited to those cases in which the cultures can be made by one who is an expert in this line of work.

The vagina and uterus being normally sterile, Marx argues that in the healthy woman the only part of the genital tract needing preparation for labor is the vulva. When the asepsis of the operator cannot be questioned no uterine or vaginal douche is necessary or advisable either before, during or after labor.

On the other hand, when the vaginal secretion is alkaline, profuse, malodorous and purulent, which in most cases means a gonorrheal infection, profuse and repeated douches are given before labor, and when labor sets in, if the discharge is still not normal, surgical scrubbings, such as are done before a major vaginal operation are indicated. When in these pathological cases labor is prolonged, vaginal douches are administered at short intervals.

He does not believe in the use of gloves, depending instead on thorough hand disinfection.

Marx believes that even in cases ot known infection uterine douching is of no service in eliminating infection, and he instead advocates the firm packing of the uterus with iodoform gauze.

